# Supplementary material for: Transcriptome Analysis Reveals Vimentin-Induced Disruption of Cell–Cell Associations Augments Breast Cancer Cell Migration
Source: Cells. 2022 Dec 13;11(24):4035. doi: 10.3390/cells11244035 (PMC9776984; doi:10.3390/cells11244035)
Supplement: Supplementary file 1 [file cells-11-04035-s001.zip › DEGs and enrichment analysis.zip!/DEGs and enrichment analysis/WT_VIMvsVector GO enrichment/ALL/WT_VIMvsVector_all.CCbar.pdf]

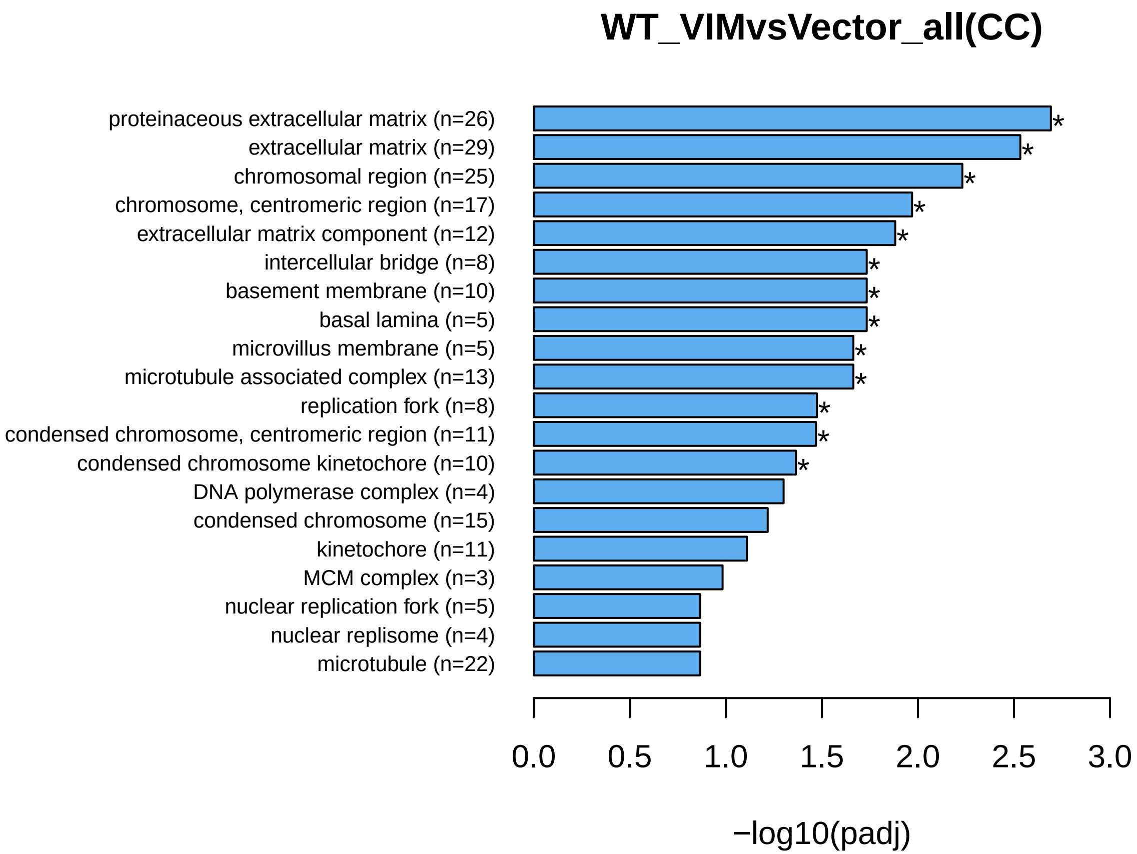
WT_VIMvsVector_all(CC)
proteinaceous extracellular matrix (n=26)
extracellular matrix (n=29)
chromosomal region (n=25)
chromosome, centromeric region (n=17)
extracellular matrix component (n=12)
intercellular bridge (n=8)
basement membrane (n=10)
basal lamina (n=5)
microvillus membrane (n=5)
microtubule associated complex (n=13)
replication fork (n=8)
condensed chromosome, centromeric region (n=11)
condensed chromosome kinetochore (n=10)
DNA polymerase complex (n=4)
condensed chromosome (n=15)
kinetochore (n=11)
MCM complex (n=3)
nuclear replication fork (n=5)
nuclear replisome (n=4)
microtubule (n=22)
0.0
0.5
1.0
1.5
2.0
2.5
3.0
−log10(padj)
